# Correlation of Enzymatic Depolymerization Rates with the Structure of Polyethylene-Like Long-Chain Aliphatic Polyesters

**DOI:** 10.1021/acsmacrolett.4c00463

**Published:** 2024-09-11

**Authors:** Simon T. Schwab, Leonie Y. Bühler, David Schleheck, Taylor F. Nelson, Stefan Mecking

**Affiliations:** † Chair of Chemical Materials Science, Department of Chemistry, University of Konstanz, Universitätsstraße 10, 78457 Konstanz, Germany; ‡ Microbial Ecology and Limnic Microbiology, Department of Biology, University of Konstanz, Universitätsstraße 10, 78457 Konstanz, Germany

## Abstract

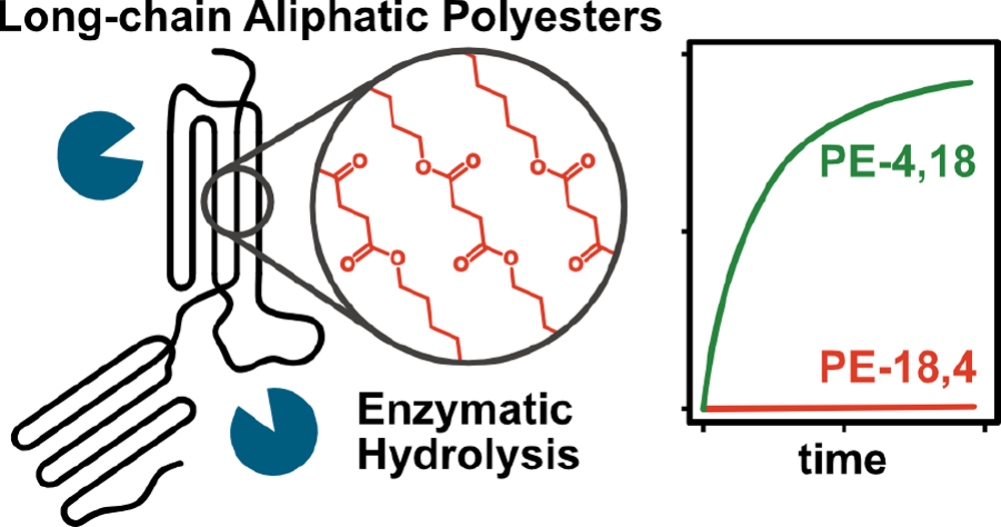

Long-chain aliphatic polyesters are emerging sustainable materials that exhibit polyethylene-like properties while being amenable to chemical recycling and biodegradation. However, varying polyester chemical structures results in markedly different degradation rates, which cannot be predicted from commonly correlated bulk polyester properties, such as polymer melting temperature. To elucidate these structure–degradability relationships, long-chain polyesters varying in their monomer composition and crystallinity were subjected to enzymatic hydrolysis, the rates of which were quantified via detection of formed monomers. Copolymers with poorly water-soluble, long-chain diol monomers (e.g., 1,18-octadecanediol) demonstrated strongly reduced depolymerization rates compared to copolymers with shorter chain length diol monomers. This was illustrated by, e.g., the 20× faster hydrolysis of PE-4,18, consisting of 1,4-butanediol and 1,18-octadecanedicarboxylic acid monomers, relative to PE-18,4. The insoluble long-chain diol monomer released upon hydrolysis was proposed to remain attached to the bulk polymer surface, decreasing the accessibility of the remaining ester bonds to enzymes for further hydrolysis. Tuning of polyester crystallinity via the introduction of branched monomers led to variable hydrolysis rates, which increased by an order of magnitude when crystallinity decreased from 72% to 45%. The results reported enables the informed design of polyester structures with balanced material properties and amenability to depolymerization.

From the early days of polymer science and technology, the hydrolytic stability of polymers has been an essential parameter. Hydrolytic depolymerization rates are controlled in the first place by the chemistry of in-chain functional groups like, e.g., esters, amides, or anhydrides. In particular, polyesters have repeatedly proven effective as materials that are environmentally degradable and chemically recyclable, dependent on their propensity to hydrolytic depolymerization.^1−5^ Therefore, understanding the controls on polyester hydrolysis is critical for determining their suitability for applications where these end-of-life options are applicable.

Polyester depolymerization is often dependent on the action of enzymes, both in engineered systems for chemical recycling under mild conditions and in the natural environment where hydrolysis by extracellular enzymes is understood to be the rate-limiting step for polyester biodegradation.^6,7^ As depolymerization catalysts, enzymes rely on the accessibility of ester bonds and, thus, are highly dependent on the solid-state structure of the substrates. They act on the amorphous regions preferentially or virtually exclusively, prominently illustrated by the need to transform polyethylene terephthalate (PET) into an amorphous state for efficient enzymatic chemical recycling.^8^ Therefore, bulk crystallinity is often recognized as a major controlling factor of hydrolytic breakdown rates.^9−11^ The crystalline and amorphous phases of polymers can be controlled by thermal and mechanical treatment, a topic being closely investigated for PET. A high shear force in PET melts leads to more crystalline materials, which in turn show reduced enzymatic hydrolysis rates.^12^ By incubating PET at temperatures close to the glass transition temperature (*T*_g_), its’ amorphous phases become more ordered and less accessible, reducing in turn enzymatic hydrolysis rates.^13^

Even with a consistent thermal history, in comparisons of different polyesters to better understand how structural features impact enzymatic hydrolysis rates, most often several parameters like crystal structure, ester group density, and hydrophobicity vary strongly at the same time, complicating the picture.^6,14,15^ The specific influence of crystallinity on degradability has so far been mostly investigated for polyesters with high *T*_g_s, including PET or PEF (polyethylene furanoate), for which crystallinity can be adjusted by quenching of a polymer melt, i.e., without changing the chemical structure of the polyesters.^16−18^ In comparison, low-*T*_g_, long-chain aliphatic polyesters maintain high-density polyethylene (HDPE)-like orthorhombic crystal structures over a range of different monomer repeat unit combinations, with ester groups located both in amorphous and crystalline regions.^19−24^ Further, they are also relevant materials in that the HDPE-like structures result in excellent mechanical properties and processability, and at the same time, the ester groups in the polyethylene chains enable chemical recycling.^25,26^

Small changes in the microstructure of long-chain aliphatic polyesters can lead to drastic changes in their hydrolytic degradation behavior without affecting bulk thermal and physical properties, as described above. As a prominent example, PE-18,18 and PE-2,18 exhibit only slight differences in crystallinity (both approximately 70%) and melting point (99 and 96 °C, respectively).^26^ Even so, these long-chain aliphatic polyesters show slow hydrolysis^27,28^ compared with that of shorter-chain aliphatic polyesters. PE-18,18 exhibits no base- or enzyme-catalyzed depolymerization at ambient conditions and limited biodegradation in industrial composting experiments.^25,26^ On the other hand, PE-2,18 is enzymatically degradable and fully compostable. Furthermore, accelerated enzymatic depolymerization and composting has been reported for other long-chain polyesters with reduced crystallinities induced via cross-links or branching.^19,29^

We now report a systematic and quantitative comparison of enzymatic hydrolysis rates of long-chain aliphatic polyesters and elucidate the influence of the microstructure on their hydrolytic degradability.

Two approaches were pursued to determine the polymer structural factors influencing the enzymatic hydrolysis of long-chain polyesters. First, the polymers’ crystallinity was tailored by a copolymerization of linear C_18_ diol and dicarboxylate monomers with a branched, biobased fatty acid dimer diol (Scheme 1a). Second, the chain lengths of linear monomers were varied to yield a set of different long- and short-long linear polyesters (Scheme 1b). All polyesters were synthesized via melt polycondensation according to published protocols.^22,23^

**Scheme 1:**
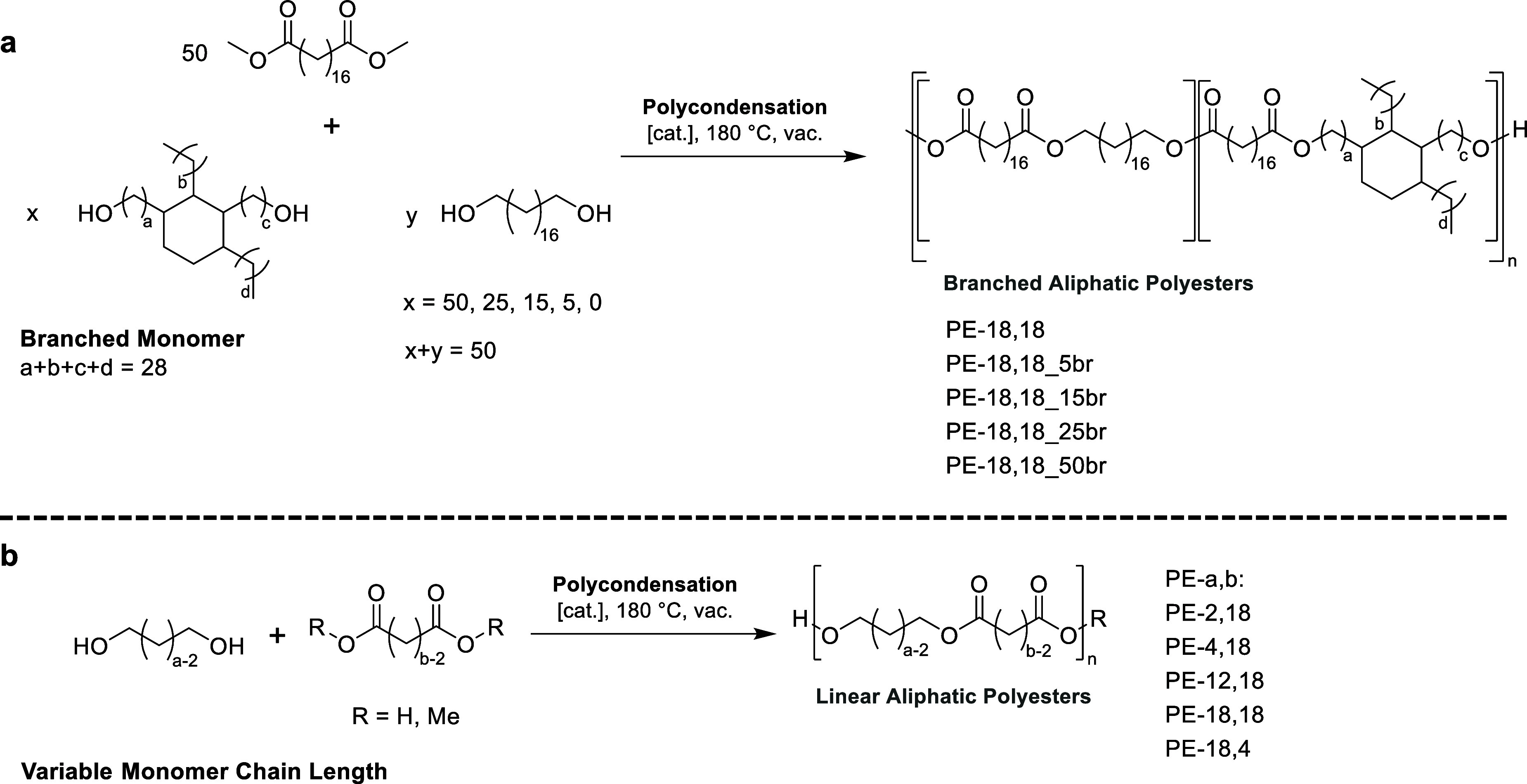
Synthesis of (a) Branched and (b) Linear Polyesters

Increasing the amount of the branched diol relative to the linear one (with the polyesters labeled as PE-18,18_*X*br, where *X* is the overall molar percent of the branched diol in the polyester) led to a systematic decrease in melting points and melting enthalpies, as well as volume crystallinity and crystallite sizes, while maintaining the orthorhombic, polyethylene-like crystal structure (see Figure S14 and Table S2). The lower degrees of crystallinity were reflected in the polymers’ mechanical properties (lower Young’s moduli and yield stresses, see Tables 1 and S3 and Figures S15–S18), yielding polymers rather resembling low-density polyethylene (LDPE) in terms of tensile properties and degrees of crystallinity. Surface free energies (SFE) remained within a range (30.2–33.7 mN m^–1^) similar to the published value for HDPE (32.4 mN m^–1^).^26^ For hydrolysis experiments, branched polyesters containing up to 25 mol % branched diol monomer were employed due to their sufficiently high melting points (>70 °C) in comparison to the hydrolysis experiment temperature (37 °C).

**Table 1:** Selected Properties of the Investigated Polyesters

polymer	*T*_m_ [°C]^a^	crystallinity [%]^b^	SFE [mN·m^–1^]^c^
PE-18,18	99	72	32.6 ± 0.4
PE-18,18_5br	96	63	33.7 ± 0.7
PE-18,18_15br	84	52	30.2 ± 2.2
PE-18,18_25br	74	45	31.2 ± 2.1
PE-2,18	96	71	36.5 ± 0.9
PE-4,18	85	71	35.6 ± 0.9
PE-12,18	93	76	33.7 ± 0.6
PE-18,4	92	72	36.4 ± 1.4

a Determined from the second heating trace in DSC (10 K min^–1^).

b Determined from WAXS diffractograms.

c Determined via contact angle measurements using H_2_O and CH_2_I_2_.

Linear polyesters PE-*a*,*b* varying in the constituent diol and diacid monomer chain lengths *a* and *b* (−[O(CH_2_)_*a*_OOC(CH_2_)_(*b*−2)_COO]_*n*_–, with *a* = 2, 4, 12, and 18 and *b* = 4 and 18), respectively, exhibited melting points between 85 and 99 °C and similar volume crystallinity (71–76%). The SFE of the polyesters varied to a limited extent depending on the ester group density in the chain, with the polyester PE-18,18 having the lowest SFE of 32.6 mN m^–1^ and PE-2,18 having the highest SFE of 36.5 mN m^–1^ (Tables 1 and S3).

Melt-pressed films (diameter 1 cm, typical weight 5 mg, typical thickness 70 μm) of all polyesters were subjected to enzymatic hydrolysis using the naturally occurring enzyme *Humicola insolens* Cutinase (HiC).^30^ Monomer formation was quantified at different times of exposure to HiC using high-performance liquid chromatography equipped with a refractive index detector (HPLC-RI, for ethylene glycol, succinic acid, and butane diol) or liquid chromatography–mass spectrometry (LC-MS, for C_18_ diacid) to determine hydrolysis rates. Polyester hydrolysis amounts are reported below as g m^–2^ for comparability between polymers and were calculated from the measured concentrations of the detected monomers and their contents in the overall polymers (see SI). All measured films stayed intact during the degradation experiments, with a maximum depolymerization extent of 63 wt % for PE-4,18 after 19 d.

Enzymatic hydrolysis experiments of the branched polyesters revealed a clear relationship between degradation and crystallinity (Figures 1 and S34). The monomer formation after 5 days for PE-18,18_25br, the least crystalline polymer, exceeded the monomer formation of PE-18,18 by more than an order of magnitude. The trend of decreasing monomer formation with increasing crystallinity was consistent across all four investigated polymers.

**Figure 1:**
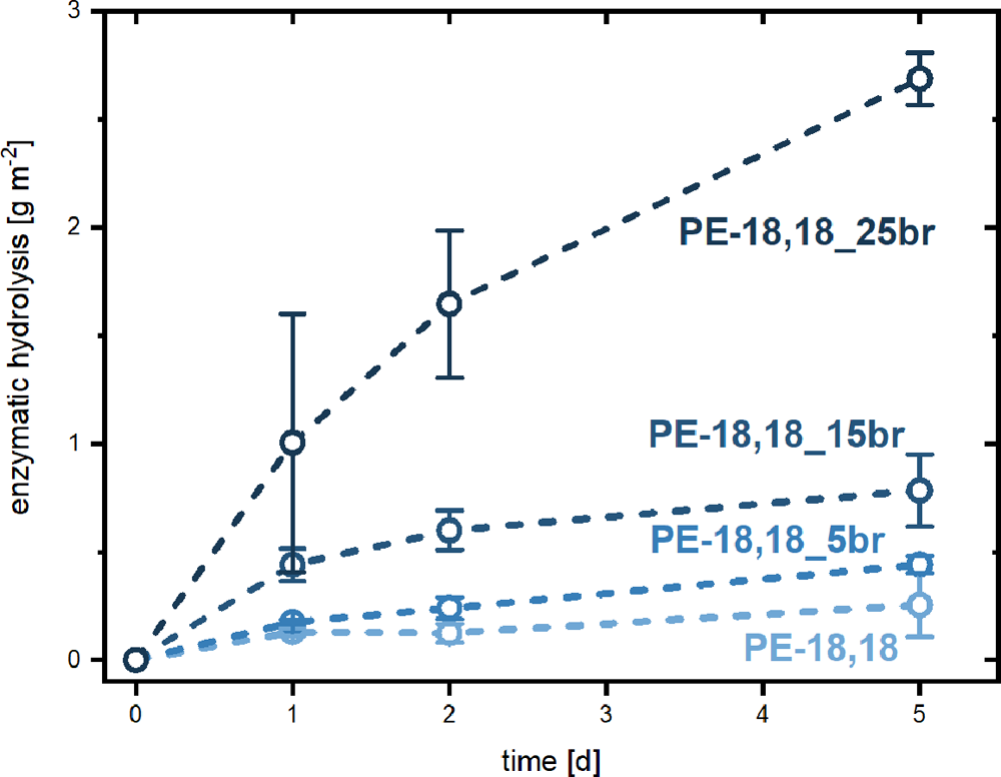
Enzymatic hydrolysis of branched aliphatic polyester films incubated in phosphate buffer (pH = 8.5) with *Humicola insolens* Cutinase at 37 °C. Amounts of degradation were quantified by determinations of C_18_-dicarboxylate monomer concentrations using LC-MS. The enzymatic hydrolysis of PE-18,18_25br after 5 days (2.7 g m^–2^) corresponds to a hydrolysis of 18 wt % of the overall material.

Enzymatic hydrolysis experiments were separately carried out for the linear aliphatic polyesters with varying monomer chain lengths in the same setup using HiC. The following trend in enzymatic hydrolysis rates was observed: PE-4,18 > PE-2,18 > PE-12,18 ≈ PE-18,4 > PE-18,18 (Figure 2 and Table S3).

**Figure 2:**
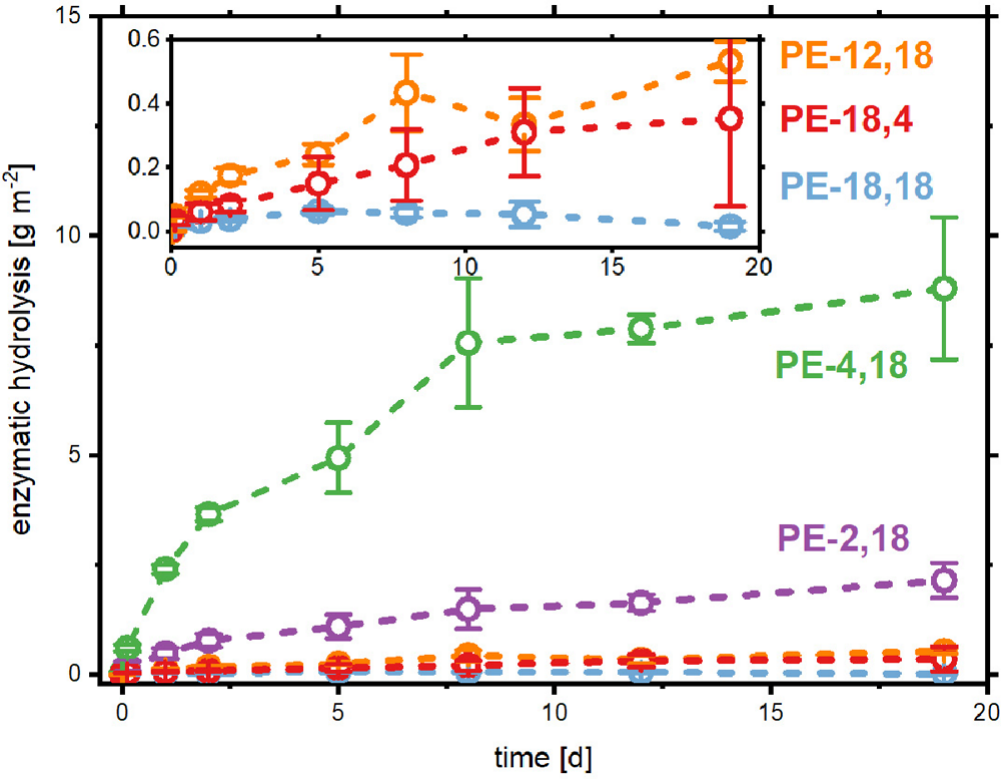
Enzymatic hydrolysis by *Humicola insolens* Cutinase of linear aliphatic polyester films at 37 °C in phosphate buffer (pH = 7.2). Amounts of degradation were determined by quantification of monomers using LC-MS or HPLC-RI, depending on the monomer detected.

The overall similar solid-state properties and microstructure of the investigated polyesters allowed the isolation of specific parameters controlling the hydrolysis rates. The enzymatic hydrolysis rates for branched polyesters increased with decreasing crystallinity (from χ ≈ 72 to 45%) by one order of magnitude. While this general trend was expected and widely reported in literature,^10,11^ it was more pronounced compared to high-*T*_g_ polymers like PET or PEF.^17,18^ This could indicate that ester bonds in amorphous phases of the here-investigated low *T*_g_ polyesters are particularly accessible for enzymes to hydrolyze compared to those in the crystalline phases. The low ester bond density and surface free energy did not impede the enzymatic hydrolysis and as such do not appear to account for the slow hydrolysis of, e.g., PE-18,18. However, strong decreases in crystallinity for the polyesters tested here were required to induce pronounced accelerations in their enzymatic depolymerization.

The enzymatic hydrolyzability of the linear polyesters was highly dependent on their monomer composition, more so than on their solid-state properties. Notably, the inclusion of long-chain diol monomers (C_12_ or C_18_) resulted in significantly reduced hydrolysis amounts, which was not the case for long-chain diacids in short-long polyesters (e.g., PE-4,18). This is clearly illustrated by the very low hydrolysis amount of PE-18,4 (0.4 ± 0.3 g m^–2^) compared to that of PE-4,18 (8.8 ± 1.6 g m^–2^) after 19 days. These polyesters are structural isomers and showed very similar properties, including crystallinity, melting enthalpies, and surface free energies. While PE-18,4 has a slightly higher melting temperature than PE-4,18, it has a lower melting temperature compared to PE-2,18, which was also hydrolyzed to a substantially higher amount (2.1 ± 0.4 g m^–2^ after 19 days). While differences in polyester melting temperature, which has been considered indicative for the polymer chains’ ability to detach from crystallites and thus for the accessibility of ester bonds in crystallites for enzymes to hydrolyze,^6,14^ may explain the enhanced hydrolysis of PE-4,18 compared to PE-2,18, this relationship clearly does not hold true across larger changes in the molecular structure of these linear long-chain aliphatic polyesters.

As stated above, a likely explanation for the vastly different degradation rates of PE-4,18 vs PE-18,4 and PE-2,18 vs PE-18,18 (also observed previously^26^) lies in the nature of the diol monomer. While typical commercial biodegradable polyesters (e.g., polycaprolactone, polybutylene succinate, and polylactic acid) consist of easily soluble monomers and even oligomers, long-chain monomers exhibit much lower water solubility. The linear C_18_ diacid has a reported solubility of 0.03 mg L^–1^ in water^31^ and the C_18_ diol monomer appears to be considerably less soluble than the diacid (see Table S4 and Figure S35). As already small monomers and oligomers are known to (co)crystallize with the remaining polymer during depolymerization,^10^ long-chain aliphatic monomers seem especially prone for such behavior. Such cocrystallization may result in a less efficient transport of the monomers away from the polymer’s surface and the formation of a protective layer around the polymer, decreasing the accessibility of the remaining in-chain ester bonds and thus hampering further enzymatic hydrolysis, as only the ester groups on the polyester surface can be hydrolyzed by enzymes.^11^ The solubility of the respective monomers could therefore explain the rapid degradation of PE-2,18 or PE-4,18, with the corresponding short-chain diols being fully miscible with water, as well as the intermediate hydrolysis of PE-12,18 and the very limited hydrolysis of PE-18,18. Note that the branched diol monomer is a viscous liquid at ambient conditions and therefore is not expected to be attached to the polymer surface in a way similar as long-chain linear diols are assumed to do. When comparing the different factors on the enzymatic hydrolysis within the series of investigated polymers, the presence of long-chain and thus poorly soluble diols (PE-18,4 vs PE-4,18) appears to have the strongest effect on enzymatic hydrolysis, surpassing the effect of crystallinity (PE-18,18 vs PE-18,18_25br) by a factor of 2. Studies on the base-catalyzed hydrolysis of these materials, precluding potential substrate/product specific inhibition, are instructive to corroborate the previous experiments, as they revealed similar trends, including a decelerated hydrolysis of polyesters containing the C_18_ diol monomer compared to those containing short-chain diols (see Figure S26).

These experiments highlight the complex mechanism and variety of parameters controlling polyester depolymerization. While crystallinity has a strong effect on enzymatic hydrolysis rates, the monomer composition also plays a major role, especially for water insoluble monomers, a so far mostly overlooked factor. These experiments were conducted under controlled and accelerated laboratory conditions; however, they can offer valuable insights into the expected degradation behavior of polyesters in natural settings. Previous studies have demonstrated that enzymatic degradation experiments using HiC or other enzymes, despite the artificially high enzyme concentration employed in these experiments, can qualitatively predict relative degradability via microorganisms in compost, reflecting the same marked disparities in the mineralization of PE-18,18 and PE-2,18.^26^ Thus, polyester monomer compositions should also have a strong effect on their environmental biodegradation, including the inhibiting effect of long-chain diol monomers. While the hydrolysis of polyesters proceeds via extracellular enzymes, ultimate biodegradation (e.g., mineralization) comprises an uptake of low molecular weight hydrolysis products, which may be greatly impeded for hydrolysis products (including monomers) with poor water solubilities.^32^ Furthermore, previous studies have indicated a slower biodegradation of long-chain aliphatic diols compared to, e.g., fatty acids.^33,34^ Consequently, long-chain diols might contribute to slower biodegradation of the resulting polyesters even under environmental conditions. However, the extent of this effect and whether other factors are ultimately rate determining will likely depend very much on the type of environment and is expected to be different in, e.g., soil vs marine or limnic environments.

The phenomenon of monomer solubility limiting hydrolysis and biodegradation rates and extents may not be confined to linear long-chain diols as discussed herein; for example, ultralong-chain diacids may be expected to show a similar behavior. Therefore, the findings of this study should apply more broadly to polymers with low densities of cleavable bonds as predetermined breaking points, meaning that polymers consisting of building blocks in the range of several kg mol^–1^ will most likely not undergo measurable enzymatic hydrolysis nor ultimate biodegradation. While not the focus of this work, these findings should also apply to controlled depolymerization of polyesters for enzymatic or chemical recycling.^1,2,22^ As depolymerization for these applications is currently often performed in basic aqueous media, the solubility and detachment of the monomers from the polymer’s surface need to be taken into account for the design of a recycling system back to the monomer, especially for approaches involving enzymatic hydrolysis at temperatures below the polyester melting point.

In summary, we report a quantitative analysis of the role of monomer composition and crystallinity in the enzymatic hydrolysis of polyethylene-like long-chain polyesters. The inclusion of water-insoluble long-chain diol monomers markedly decreased polyester hydrolysis rates by orders of magnitude, potentially due to the monomers’ attachment to polyester surfaces upon depolymerization, blocking further enzymatic hydrolysis. Less crystalline materials did undergo faster and more extensive hydrolysis, which was not prevented by the low ester-bond densities and nonpolar nature of these materials.

Our findings further elucidate the role of structural parameters on polyester hydrolysis in general. In particular they provide guidelines for the design of circular nonpersistent materials, with regard to enzymatic chemical recycling, as well as tuning of the hydrolytic stability for polymer applications and for environmental breakdown as a backstop for materials lost from recycling and other recovery streams.^35^

## Supporting Information Available

Experimental procedures and polymer characterization data, including polymer synthesis, degradation sample preparation, degradation experiments procedure, workup and quantification, additional polymer characterization (NMR spectra, SEC chromatograms, DSC traces, WAXS diffractograms, stress–strain curves), calibration curves, base-catalyzed hydrolysis data, and solubility data for aliphatic diacids and diols (PDF)
