# Supplementary material for: Correlation of Enzymatic Depolymerization Rates with the Structure of Polyethylene-Like Long-Chain Aliphatic Polyesters
Source: ACS Macro Lett. 2024 Sep 11;13(10):1245–50. doi: 10.1021/acsmacrolett.4c00463 (PMC11483938; doi:10.1021/acsmacrolett.4c00463)
Supplement: Supplementary file 1 — mz4c00463_si_001.pdf [file mz4c00463_si_001.pdf]

Supporting Information

for

Correlation of Enzymatic Depolymerization Rates with the Structure of Polyethylene-like Long-chain Aliphatic Polyesters

*Simon T. Schwab, Leonie Y. Bühler, David Schleheck, Taylor F. Nelson, Stefan Mecking**

Chair of Chemical Materials Science, University of Konstanz,

Department of Chemistry, Universitätsstraße 10, 78457 Konstanz, Germany.

*Stefan.mecking@uni-konstanz.de

Table of Content

Methods and Materials 1
Additional Characterization Data for Polyesters 9
Liquid chromatography calibration curves 22
Further Hydrolysis Data 24
Summary Properties and Degradation of Polyesters 26
Solubility of Long-chain Diacids and Diols 27
References 29

Methods and Materials

Unless stated otherwise all polymerizations and manipulations involving moisture and/or air sensitive substances were performed under inert atmosphere using standard Schlenk and glovebox techniques.

Materials

All chemicals were used as received without further purification. 1,18-octadecanoic acid (C_{18} diacid) was purchased from Elevance Renewable Sciences Inc. Titanium(IV) *n*-butoxide (97%), dibutyltin oxide (DBTO), para-nitrophenyl butyrate (≥ 98 %), 1,4-butanediol (99 %), 1,12-dodecanediol (99 %), and methanol (≥ 99.8 %) were purchased from Sigma Aldrich. Xylene (isomeric mixture, ≥ 99 %), ethylene glycol (EG, ≥ 99.5 %), chloroform (>90 %), and di-sodium hydrogen phosphate dihydrate (≥ 98 %) were obtained from Carl Roth. 2-Propanol (≥ 99.7 %) was purchased from VWR, 1 M aqueous sodium hydroxide solution from Merck, and succinic acid (>99 %) from TCI chemicals. For LC-MS measurements, 2-propanol (≥ 99.9 %) was purchased from Merck, formic acid (98-100 %) from Riedel-de Haën and acetonitrile (≥ 99.9 %) from VWR. Sulfuric acid (96 %), sodium dihydrogen phosphate monohydrate (≥ 99.99 %), and para-nitrophenol (≥ 97 %) were purchased from Merck. Deuterated solvents for NMR spectroscopy were purchased from Eurisotop and dried over molecular sieves from Riedel-de Haën (0.4 nm). The enzyme *Humicola insolens* cutinase (Lipase 51032, StickAway) was purchased from ChiralVision. Fatty acid dimer (Pripol™ 1009, 98 %, branched diacid) and dimer diol (Pripol™ 2033, branched diol) were kindly provided by Croda / Cargill. 1,19-Nonadecanoic acid (C_{19} diacid) was synthesized as reported previously.[1] For experiments with enzymes, Milli-Q (Millipore) water was used to prepare all solutions.

1,18-dimethyl octadecanedioate and 1,18-octadecanediol were prepared as reported previously.[2]

Characterization Techniques

Nuclear magnetic resonance (NMR) spectroscopy was performed on a Bruker Avance III HD 400 or a Bruker Avance III 400 spectrometer. Chemical shifts were referenced to the solvent signals. Mestrenova software Mestrelab Research S.L. (version 14.1.2) was used for data evaluation. Spectra of polymers were acquired in $CDCl_3$ or in $C_2D_2Cl_4$ at 300 K or 383 K, respectively, with 128 scans.

Wide angle x-ray scattering (WAXS) diffractograms were recorded on a D8 Discover Bruker instrument with a Vantec or Lynxeye detector. The crystallinity X_{WAXS} of pretreated polymer specimen (cooled from 200 °C to room temperature with 10 K/min) was determined from the WAXS diffractograms as reported previously.[3] The [110] and [200] lattice spacings *d* of the orthorhombic, HDPE-like crystal structure were determined using the Bragg equation:[4]

$$n\lambda = 2d sin\theta \qquad (1)$$

λ is the wavelength of the Cu-K_α x-ray beam (1.5406 Å), and the angle θ refers to the maximum of the diffraction peaks. Using the Scherrer equation,[5] the crystallite size normal to the planes (hkl) was calculated from the full width at half maximum of the reflections (FWHM, β_x) and the Bragg angle for the hkl planes:

$$D_{hkl} = \frac{0.89 \cdot \lambda}{\beta_x \cdot \cos(\theta)} \qquad (2)$$

Molecular masses of polymers were determined *via* size exclusion chromatography (SEC) in chloroform at 35 °C on a PSS SECcurity[2] instrument, equipped with PSS SDV linear M columns

(2 × 30 cm, additional guard column) and a refractive index detector (PSS SECcurity^2 RI). A standard flow rate of 1 mL min $^{-1}$ was used. Data was evaluated versus polystyrene standards using the software PSS WinGPC, version 8.32.

Differential scanning calorimetry (DSC) measurements were carried out on a Netzsch DSC 204 F1 instrument with Netzsch Proteus Thermal Analysis software, version 6.1.0. Data reported for melting points (T_m) are from the second heating cycle with a heating / cooling rate of 10 K min^{-1}, for glass transition temperatures (T_g) from the second heating cycle using a heating / cooling rate of 30 K min^{-1}.

Prior to injection molding, the polymers were homogenized in an Xplore MC 15 or Xplore MC 5 microcompounder at 140 °C to 160 °C for 5 min. Tensile testing specimens were injection molded with an Xplore IM 5.5 injection molder, at a cylinder temperature identical to the compounder temperature and a mold temperature of 30 – 50 °C. An injection pressure of 16 bar for 10 s and 12 bar for 15 s was applied.

Tensile tests were performed on a Zwick Z005/1446 Retroline tC II instrument at a crosshead speed of 5 mm min^{-1} on injection molded tensile tesing specimen (ISO 527-2, type 5A). The Young's modulus was determined at a crosshead speed of 0.5 mm min^{-1}. The tensile testing samples were preconditioned for a minimum of one day prior to the measurement. testXpert software from Zwick Roell, version 11.0, was used for data evaluation.

The surface free energy (SFE) of the polymers was determined on injection molded or melt-pressed samples by the method of Fowkes on a drop shape analyzer DSA25 by KRÜSS, using diiodomethane and water as liquid phase.[6]

The concentration of EG, succinic acid, and 1,4-butanediol in enzymatic hydrolysis samples were determined using a HPLC-RI with a Rezex RHM-monosaccharide H+ 300 9 7.80 mm 8 lm ion exchange column (Phenomenex), as reported previously for EG.[2] The column was operated at 40 °C with 30 mM sulfuric acid as mobile phase at a flow rate of 0.6 mL min^{-1}. For the quantification, a refractive index detector RID-20A (Shimadzu) was used and signals obtained were analyzed with the Shimadzu Lab Solutions software version 5.81. The retention time in this setup was 17.3 min for EG, 13.8 min for succinic acid, and 22.6 min for 1,4-butane diol. The individual calibration curves are shown in Figure S22 to Figure S **24**. Error bars, if shown, originate from standard deviations of triplicates.

The concentration of C_{18} diacid was determined using a LC-MS (LCMS-2020, Shimadzu) with a ODS Hypersil™ C18 column (ThermoFisher Scientific), as reported previously for EG.[2] As a mobile phase, a gradient of two solvents (A: 10 vol% acetonitrile and 0.1 vol% formic acid, filled up with MilliQ water, B: 100 % acetonitrile) was used. The gradient started with a 50 vol% concentration of B, increasing up to 95 vol% with a flow rate of 0.3 mL min^{-1}. The retention times of C_{18} and C_{19} diacid in this setup were 6.90 and 8.13 minutes, respectively. A calibration curve is shown in figure Figure S **25**. Error bars, if shown, originate from standard deviations of triplicates.

Polymerization Experiments

Polyesters were synthesized by melt polycondensation from diol and diacid or dimethylester, respectively, monomers. For polymers based on volatile monomers, namely PE-2,18 and PE-4,18, a two-fold excess of the volatile diol was employed and the polymerization was carried out as previously reported.[3] For all other polymers, based on a non-volatile diol, the monomers were employed in an equimolar ratio, according to a protocol previously reported for PE-18,18.[2] In brief,

for a typical polycondensation, the monomers and a catalyst (DBTO or Titanium-*n*-butoxide) were added to a Schlenk flask, stirred and heated up to around 180 °C, while gradually applying vacuum, to reach the final vacuum provided by the rotary vane pump (ca. 0.05 mbar) after 6 h, and polymerization was continued for another ca. 16 h. The viscous polymer melt was dissolved in xylene and precipitated in cold 2-propanol.

Sample Preparation for Enzymatic Hydrolysis Experiments

Films were prepared by compression molding with a P/O/Weber press using 0.3 g of polymer in a 9 cm diameter mold. The mold was heated up to 200 °C, the polymer was placed in the mold between two PTFE sheets and pressed with 15 kN overnight while the mold cooled down to room temperature. From the obtained melt-pressed films (thickness of around 70 µm), discs with a diameter of 1 cm were cut out, weighing typically around 5 mg.

Enzymatic and Abiotic Hydrolysis Studies

The enzymatic degradation experiments were carried out using the naturally occurring enzyme *Humicola insolens* Cutinase (HiC, Lipase 51032, StickAway). The activity of the enzyme against the model substrate *para*-nitrophenyl butyrate (pNPB) was tested using an approach adapted from Ribitsch *et al.*,[6] as described in a preceding publication.[3] The enzymatic degradation experiments were carried out using the same batch of enzyme stock solution. The activity of the enzyme stock solution was tested before using it in polyester hydrolysis experiments, and found to be unaltered over these series of experiments. The enzyme HiC was chosen as it showed high activity towards linear, long-chain aliphatic polyesters in previous studies and as degradation experiments using it could predict biodegradation of these polymers.

In polyester hydrolysis experiments, all samples were carried out in triplicates unless noted otherwise.

The enzymatic hydrolysis of linear polyesters was carried out using melt-pressed films with a diameter of 1 cm. The film was immersed into a tube with a volume of 2.5 mL together with 1 mL of the hydrolysis medium, containing phosphate buffer (pH 7.2, 50 mM) and HiC (8 mL enzyme stock solution per L of buffer), and a glass sphere with a diameter of 1 mm to ensure optimal mixing. The samples were rotated with 20 rpm at 37 °C. For sampling of the HPLC-RI samples, 100 µL of the degradation medium was sampled and added to 100 µL 0.5 M aq. H_2SO_4, and filtered using a 0.45 µm PTFE syringe filter. Two or a maximum of three samples were taken at different time points from the same vial (vial 1: 4 h, 1 d, 2 d; vial 2: 5 d, 8 d; vial 3: 12 d, 19 d). For sampling the LC-MS samples, 1 mL of 2-propanol with 0.2 mM C_{19} diacid as internal standard was added to the degradation medium and the polyester film and inverted together for at least 30 min at 37 °C, to fully dissolve the C_{18} diacid. The samples were filtered with a 0.45 µm PTFE syringe filter. In all samples, the polyester films remained structurally intact over the course of the experiment. Control samples without added enzyme solution performed in parallel showed negligible monomer formation.

For abiotic hydrolysis experiments with linear polyesters and PE-18,18_25br, four melt-pressed films with a diameter of 1 cm with a cumulative mass of 20 mg were added together with a glass sphere with a diameter of 1 mm to a glass vial with 10 mL 1 M NaOH aq. solution. The vials were shaken at 50 rpm at 37 °C and inverted at regular intervals. For collecting the HPLC-RI samples, 100 µL of the degradation medium was added to 100 µL 0.5 M aq. H_2SO_4, and filtered using a 0.45 µm PTFE syringe filter. For the sampling of the LC-MS, 100 µL of the degradation medium was added to 400 µL of 2-propanol with 0.2 mM C_{19} diacid as internal standard, subsequently

500 µL of phosphate buffer with a pH of 7.2 and 100 µL 1 M HCl solution (aq.) were added additionally, to ensure a full solubility of the C_{18} diacid and a neutral pH of around 7.2. Note that the solubility of the C_{18} dicarboxylate is higher by an order of magnitude compared to the C_{18} diacid in neutral aqueous conditions. The resulting solution was filtered and measured *via* LC-MS. All films remained structurally intact over the course of the experiment.

The enzymatic degradation experiments of branched polyesters containing the branched diol monomer were carried out as the enzymatic degradation experiments of linear polyesters described above with the only difference that in the degradation medium a pH of 8.5 was utilized instead of a pH of 7.2 as described above.

In previous studies, it was shown that HPLC-RI and LC-MS have a good compatibility and give the same results for the same degradation studies, even when detecting different monomers. This was also confirmed during these studies.

The amount of hydrolysis was calculated from the measured concentration of the monomers, their contribution to the overall composition of the polymer studied, the volume of the degradation medium, and the surface area of the polymer films. To this end, the amount of released monomer at each time point was multiplied with the molar mass of the repeat unit of the relevant polymer (see **Table S1**) and divided by the initial surface area of the degraded film (calculated as two times the cut-out disc area), yielding surface area normalized hydrolyzed masses of monomer (g m^{-2}). For the branched polyesters, comprised of two different diol-based repeat unit, the average mass of a [-OOC$(CH_2)_{16}$COO-R-] repeat unit taking into account the composition and mass of the diols was employed.

Table S1: Molar masses of the repeat unit of investigated polyesters.

Polymer	Molar Mass of Repeat Unit [g mol^{-1}]
**PE-2,18**	340.5
**PE-4,18**	368.6
**PE-18,4**	368.6
**PE-12,18**	480.8
**PE-18,18**	564.9
**PE-18,18_5br**	590.5
**PE-18,18_15br**	641.6
**PE-18,18_25br**	692.8

Additional Characterization Data for Polyesters

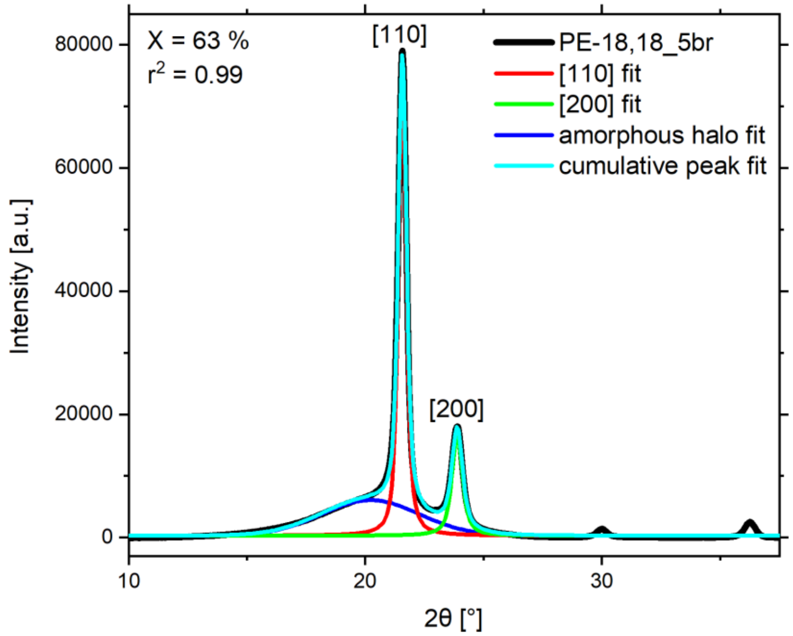

Figure S1: WAXS diffractogram of PE-18,18_5br.

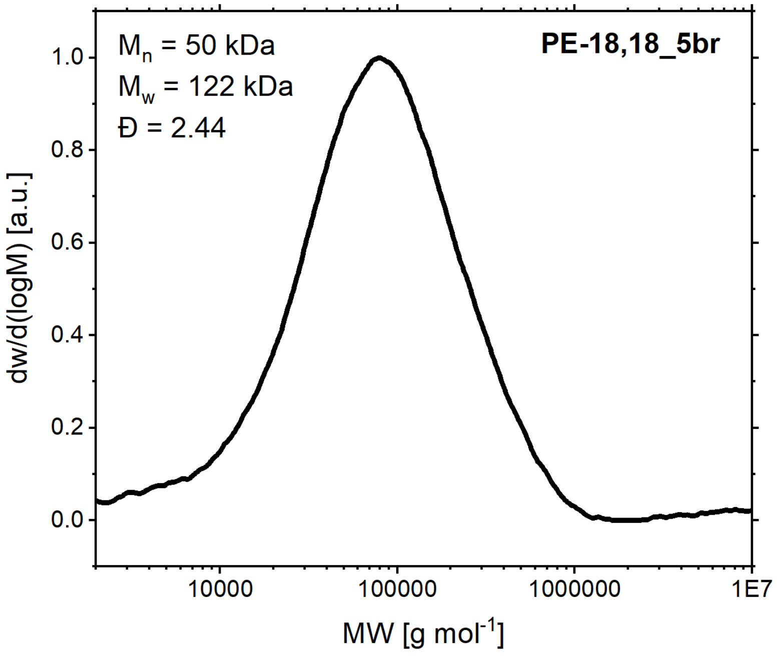

Figure S2: SEC chromatogram of PE-18,18_5br.

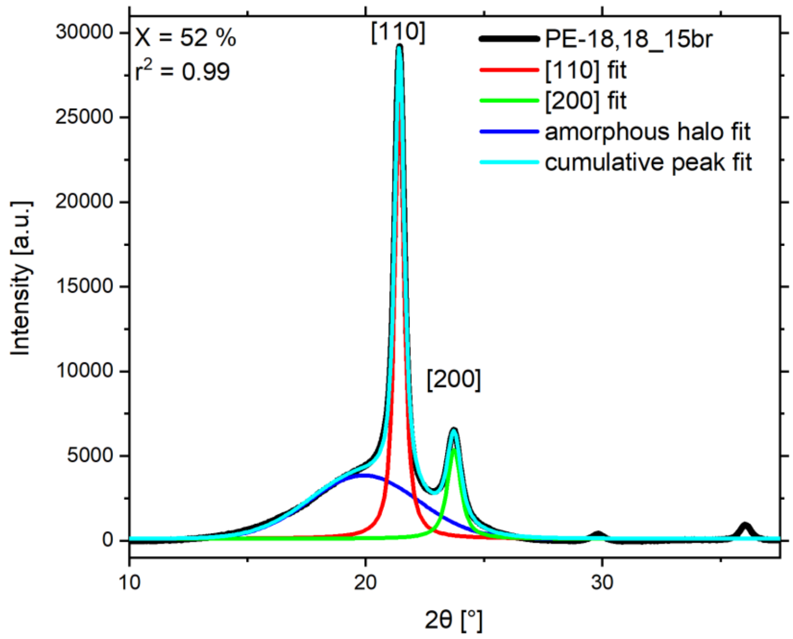

Figure S3: WAXS diffractogram of PE-18,18_15br.

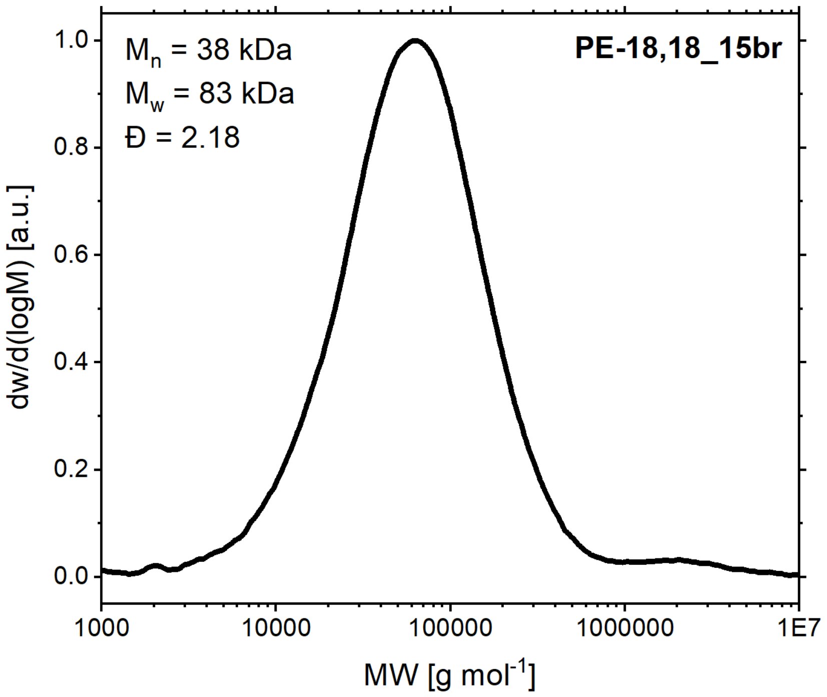

Figure S4: SEC chromatogram of PE-18,18_15br.

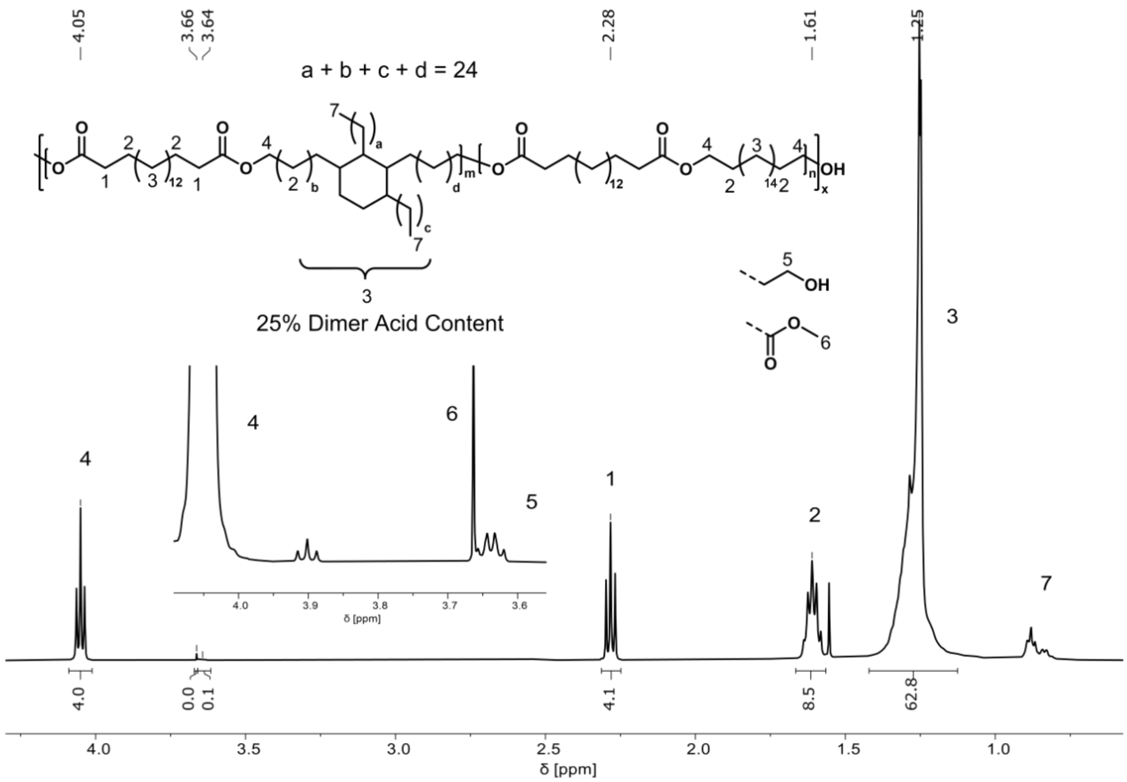

Figure S5: ^{1}H NMR spectrum (400 MHz, $CDCl_3$, 300 K) of PE-18,18_25br as exemplary NMR spectrum for linear-branched copolymers.

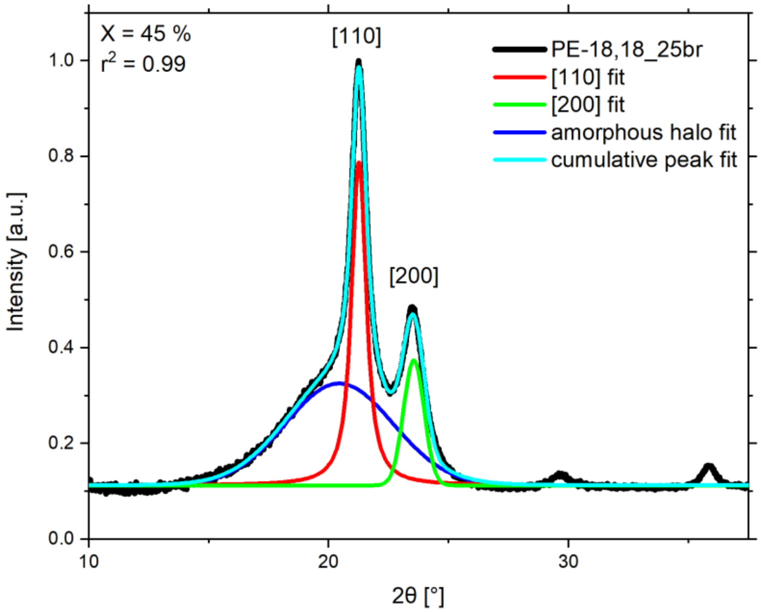

Figure S6: WAXS diffractogram of PE-18,18_25br.

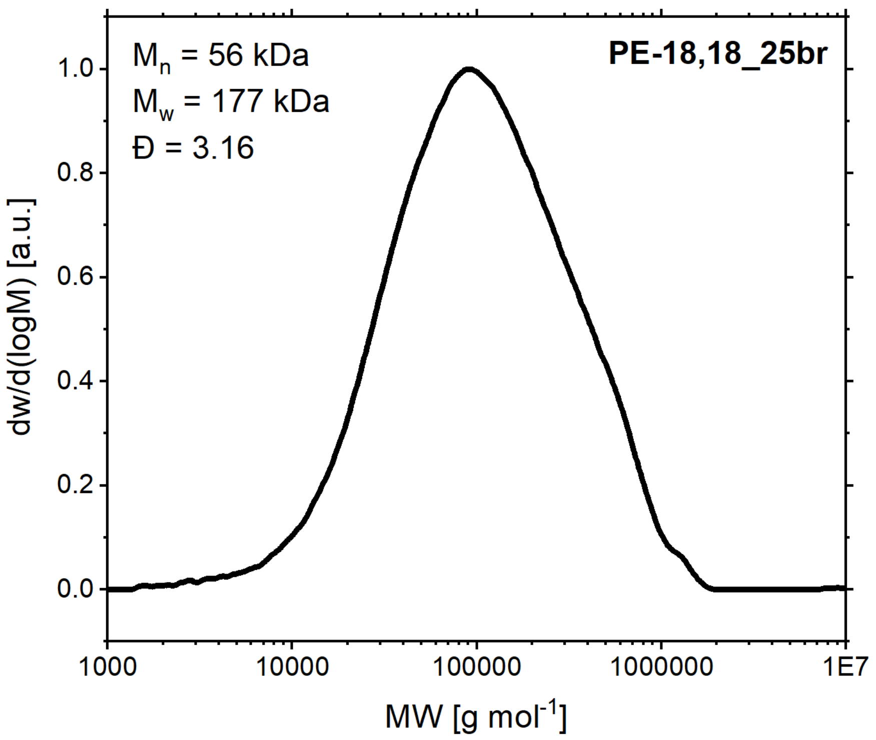

Figure S7: SEC chromatogram of PE-18,18_25br.

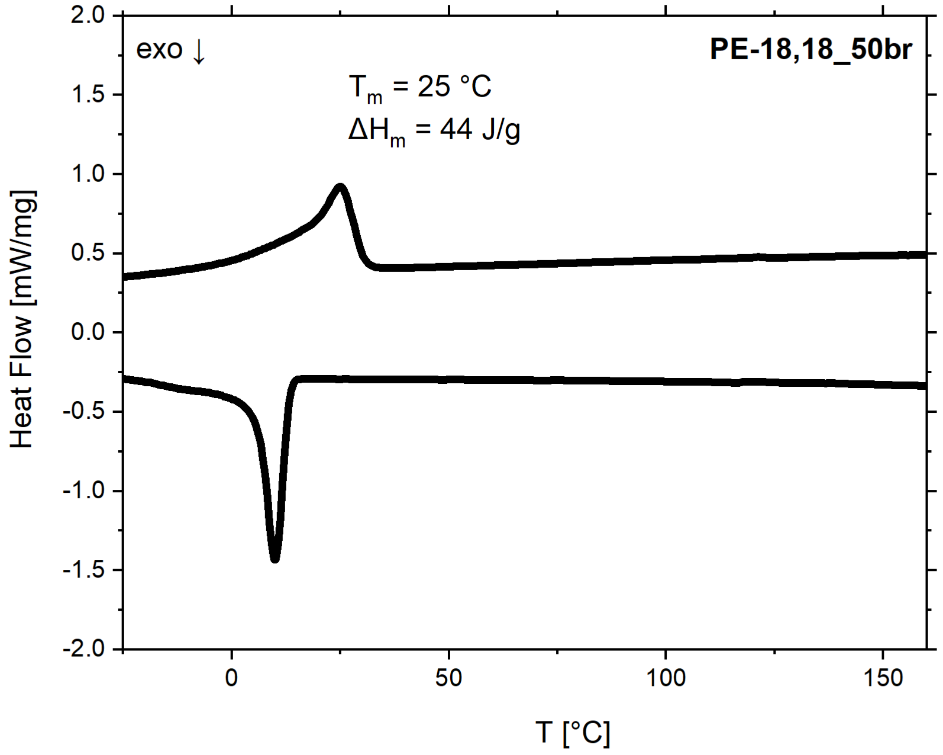

Figure S8: DSC trace of PE-18,18_50br.

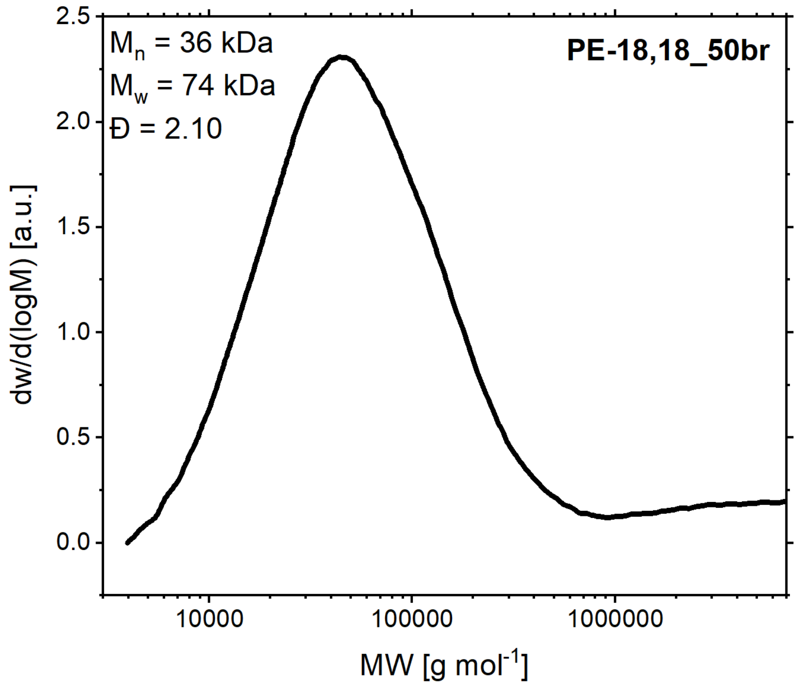

Figure S 9: SEC chromatogram of PE-18,18_50br.

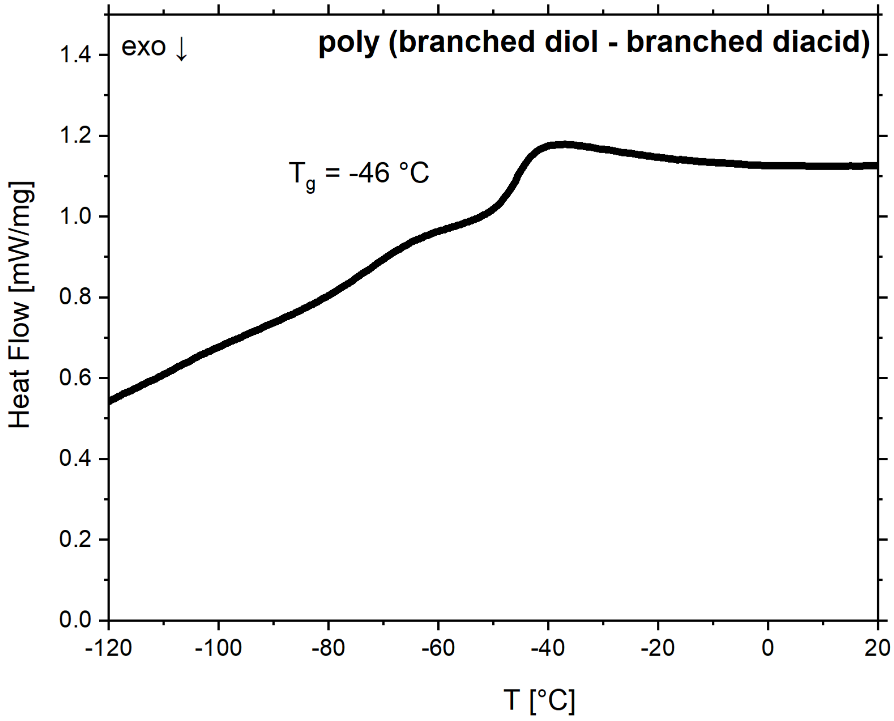

Figure S 10: DSC trace of poly(branched diol – branched diacid).

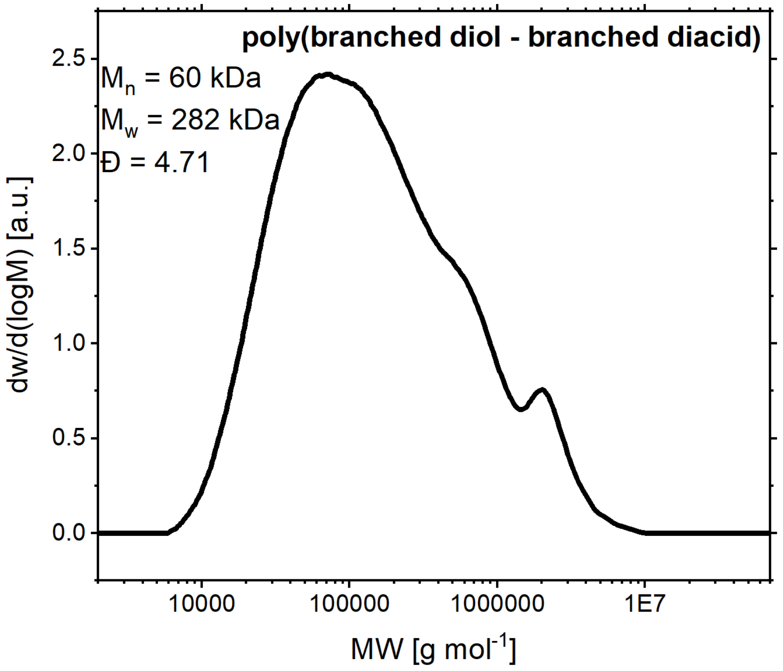

Figure S11: SEC chromatogram of poly(branched diol – branched diacid).

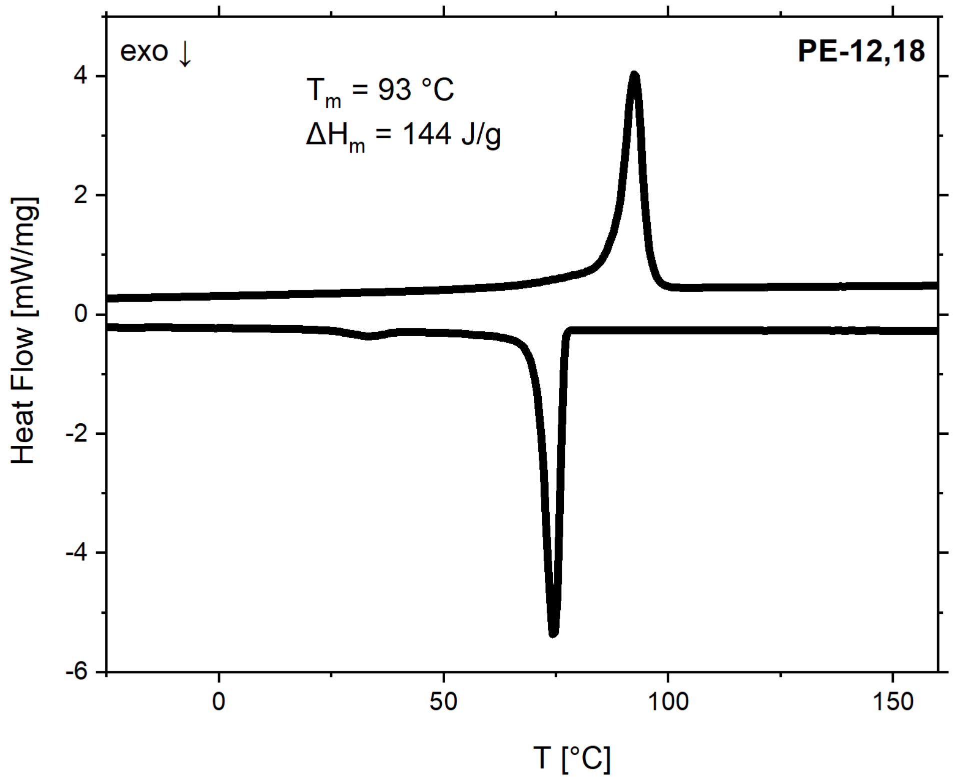

Figure S12: DSC trace of PE-12,18.

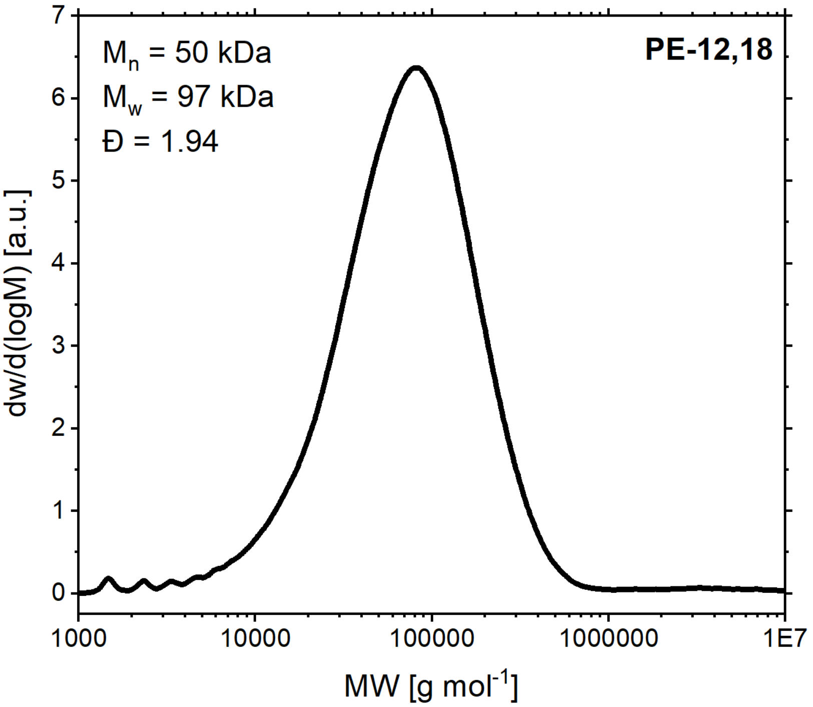

Figure S13: SEC chromatogram of PE-12,18.

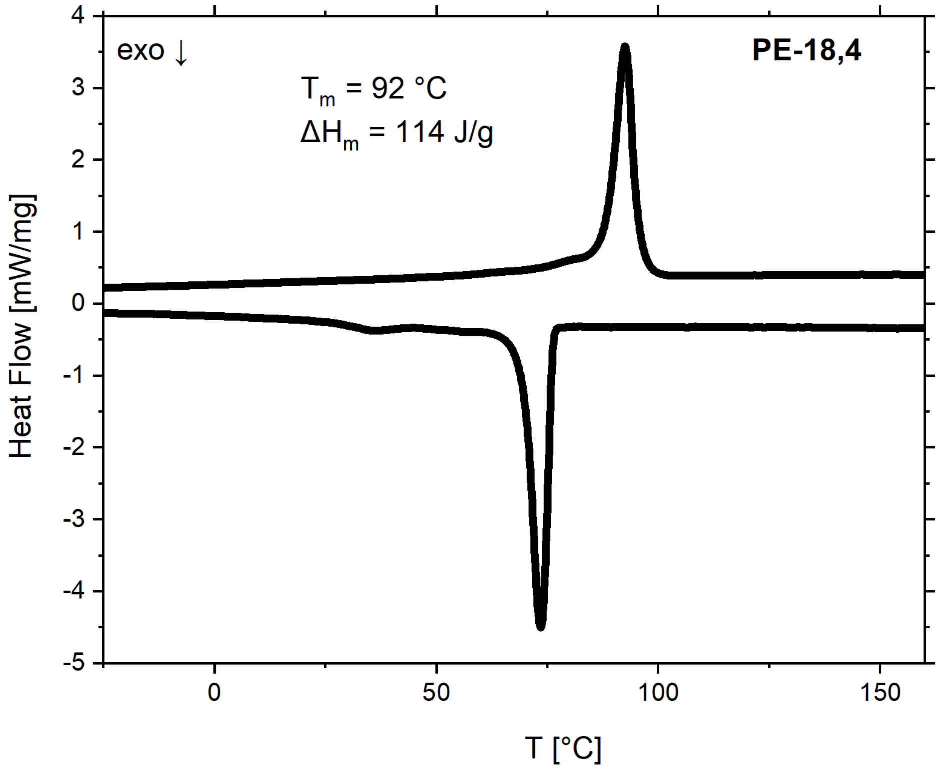

Figure S14: DSC trace of PE-18,4.

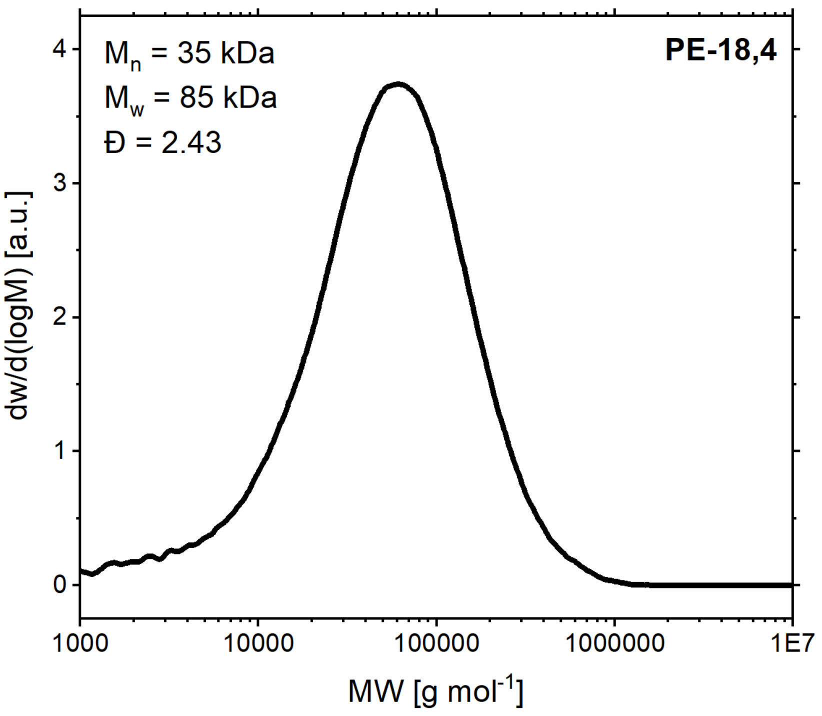

Figure S15: SEC chromatogram of PE-18,4.

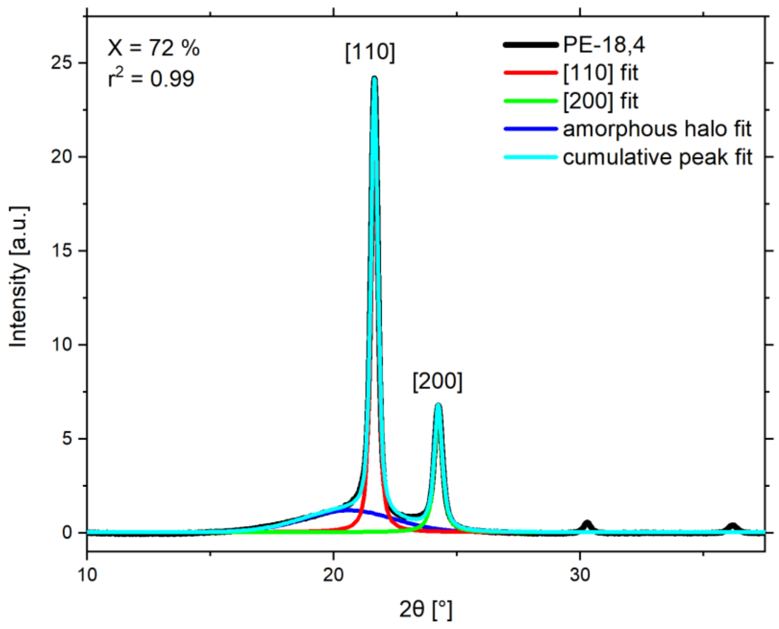

Figure S16: WAXS diffractogram of PE-18,4.

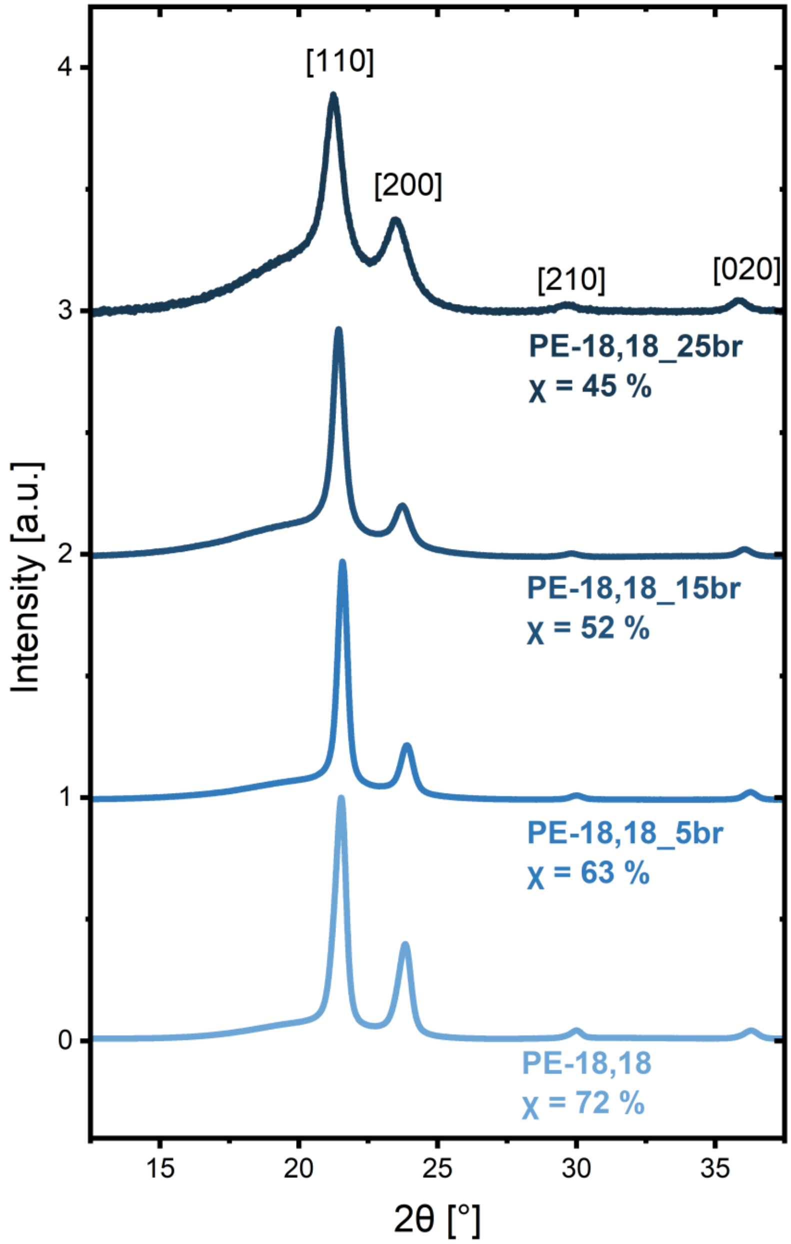

Figure S17: WAXS diffractograms of branched polyesters.

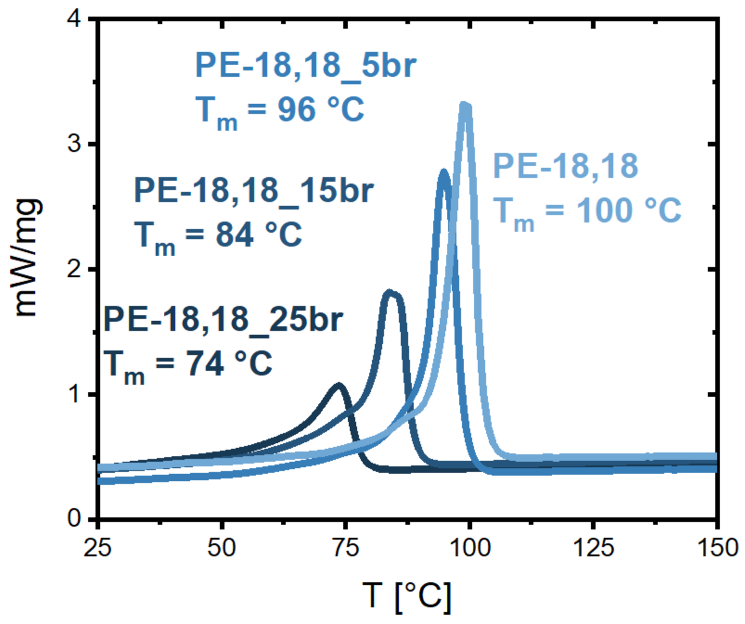

Figure S18: DSC of branched long-chain polyesters.

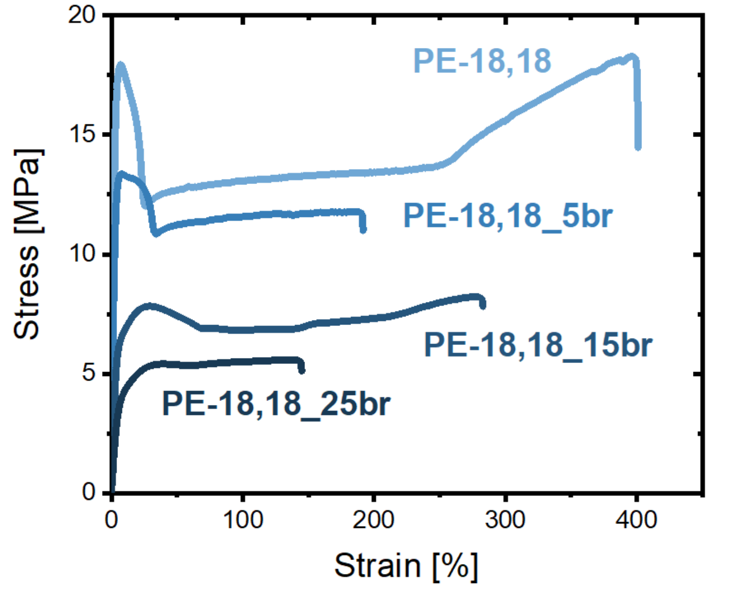

Figure S19: Stress-strain diagrams of branched long-chain polyesters.

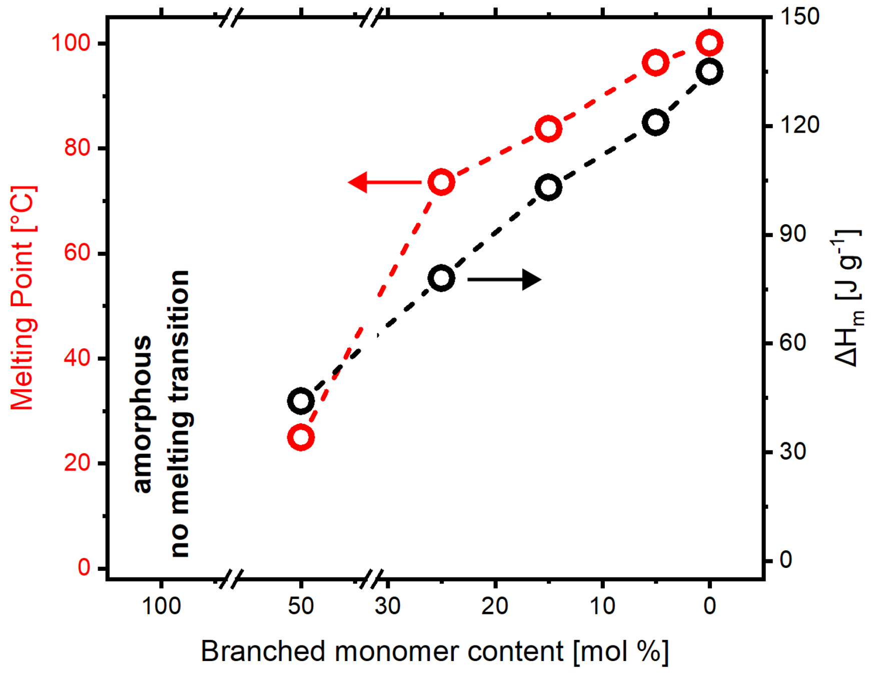

Figure S20: T_m and melting enthalpy vs. the branched comonomer content in PE-18,18. Dotted connecting lines are merely a guide to the eye.

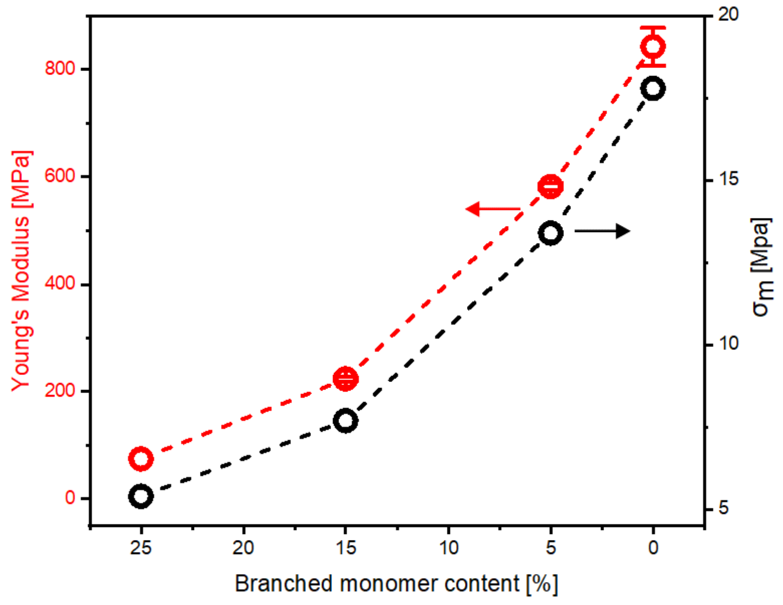

Figure S21: Young's modulus and stress at yield vs. branched comonomer content in PE-18,18. Dotted connecting lines are merely a guide to the eye.

Table S2: Crystallinity, distance of the [110] and [200] planes and crystallite sizes of branched polyesters.

Polymer	χ [%]	d [nm]		Crystallite Size [nm]	
		[110]	[200]	[110]	[200]
PE-18,18	72	41.2	37.3	16.2	24.6
PE-18,18_5br	63	41.2	37.3	18.1	21.4
PE-18,18_15br	52	41.4	37.5	13.8	10.5
PE-18,18_25br	45	41.8	37.8	8.16	9.44

Liquid Chromatography Calibration Curves

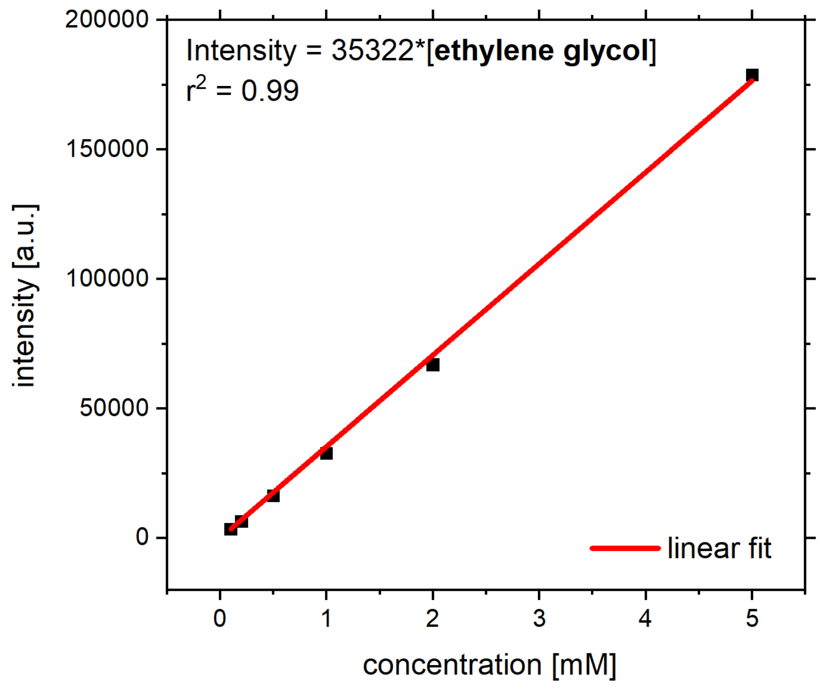

Figure S22: HPLC-RI calibration curve for EG, including the linear fit.

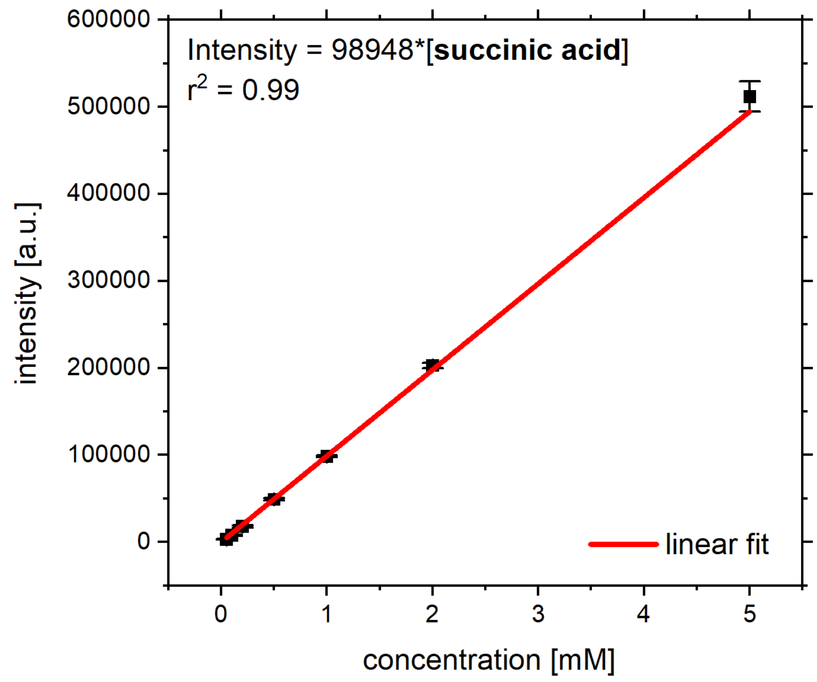

Figure S23: HPLC-RI calibration curve for succinic acid, including the linear fit.

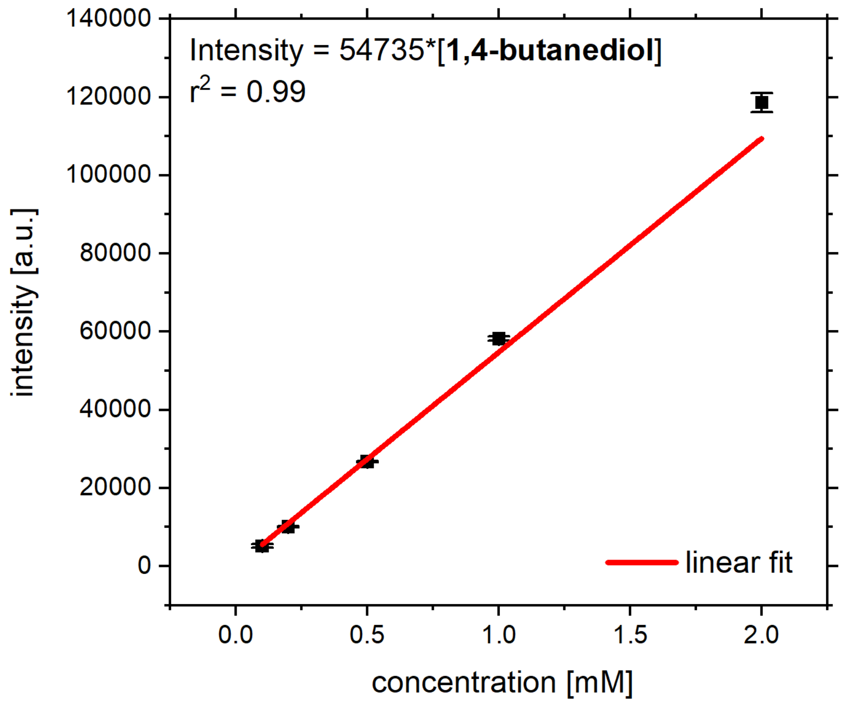

Figure S 24: HPLC-RI calibration curve for 1,4-butane diol, including the linear fit.

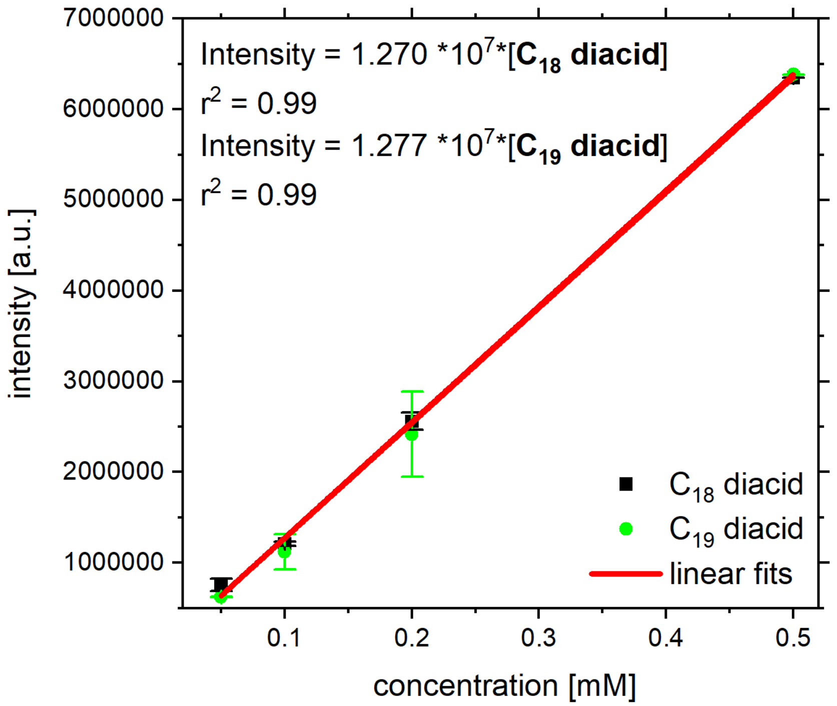

Figure S 25: LC-MS calibration curves of C_{18} and C_{19} diacid, including their linear fits.

Further Hydrolysis Data

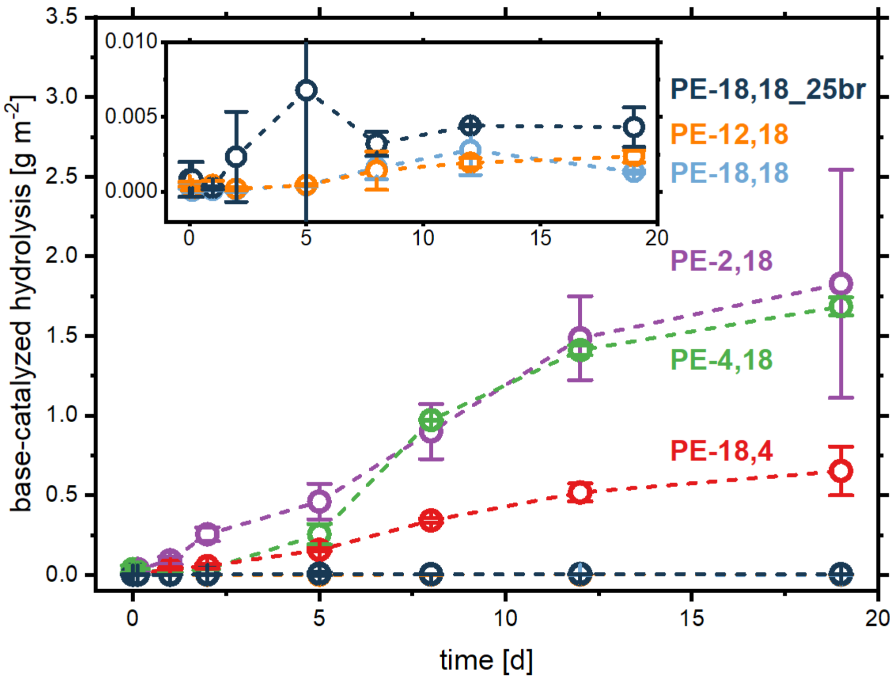

Figure S26: Base-catalyzed hydrolysis of films of linear aliphatic polyesters and PE-18,18_25br in 1 M NaOH at 37 °C over 19 d.

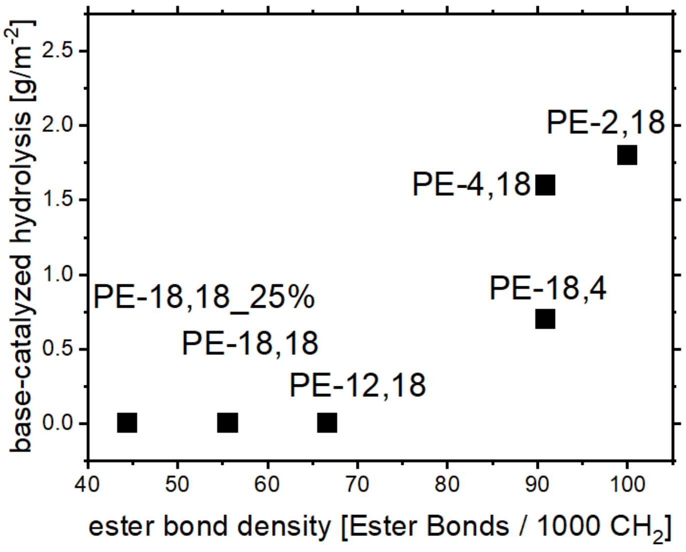

Figure S27: Abiotic degradation after 19 d vs. the ester bond density.

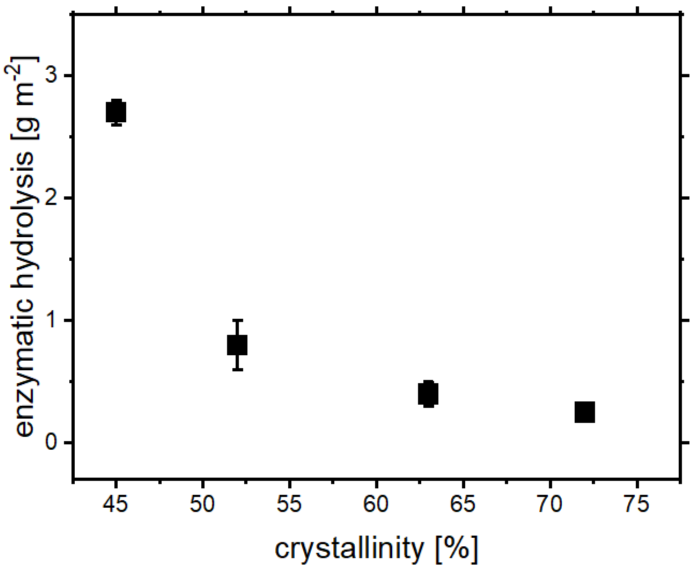

Figure S28: Enzymatic degradation of films of branched polyesters and the linear reference using the naturally occurring enzyme *Humicola insolens* Cutinase at 37 °C with a pH of 8.5 after 5 d vs. the crystallinity of the samples.

Summary Properties and Degradation of Polyesters

Table S3: Summary of properties and degradation rates of investigated polyesters.

Polymer	M_n [kg mol^{-1}]	M_w [kg mol^{-1}]	T_m [°C]	ΔH_m [J g^{-1}]	Crystall-inity [%]	SFE [mN*m^{-1}]	Ester bond density [Ester bonds / 1000 C]	Degradation [g m^{-2}], 5 d, Enzyme	Degradation [g m^{-2}], 19 d Enzyme	Degradation [g m^{-2}], 19 d NaOH
**PE-2,18**	70	126	96	116	71	36.5 ± 0.9	100	1.1 ± 0.3	2.1 ± 0.4	1.8 ± 0.7
**PE-4,18**	47	105	85	116	71	35.6 ± 0.9	91	4.9 ± 0.8	8.8 ± 1.6	1.6 ± 0.1
**PE-12,18**	50	97	93	144	76	33.7 ± 0.6	67	0.2 ± 0.03	0.5 ± 0.06	0.002 ± 0.0004
**PE-18,18**	53	123	99	144	72	32.6 ± 0.4	56	0.06 ± 0.01*	0.05 ± 0.04	0.001 ± 0.0001
**PE-18,4**	35	85	92	114	72	36.4 ± 1.4	91	0.2 ± 0.1	0.4 ± 0.3	0.7 ± 0.2
**PE-18,18_25br**	56	177	74	78	45	31.2 ± 2.1	44	2.7 ± 0.1	-	0.004 ± 0.001
**PE-18,18_15br**	38	83	84	103	52	30.2 ± 2.2	48	0.8 ± 0.2	-	-
**PE-18,18_5br**	50	122	96	129	63	33.7 ± 0.7	53	0.4 ± 0.1	-	-

* Enzymatic hydrolysis at a pH of 7.2. The amount of hydrolysis at pH = 8.5 is 0.25 g m^{-2}.

Solubility of Long-chain Diacids and Diols

Systematic data on the water-solubility of long-chain diols and diacids including specification of relevant parameters like pH of the medium[7] is scarce, potentially due to methodological challenges in determining solubilities in the range of μg L^{-1} or nmol L^{-1}. Reported data suggests that log(solubility) correlates linearly with the number of carbon atoms of the solute (Table S4 and Figure S29).

Table S4: Reported solubilities of linear aliphatic diacids and diols.

Number of Carbon Atoms in Compound	Diacid	Diol
8	11900 mg L^{-1} [8]	2538 mg L^{-1} [9]
10	1000 mg L^{-1} [10]	274 mg L^{-1} [11]
12	40 mg L^{-1} [10]	18.7 mg L^{-1} [12]
14	40.3 mg L^{-1} [13]	-
16	1.49 mg L^{-1}	-
	5.7 mg L^{-1} [14]	
18	0.03 mg L^{-1} [10]	-

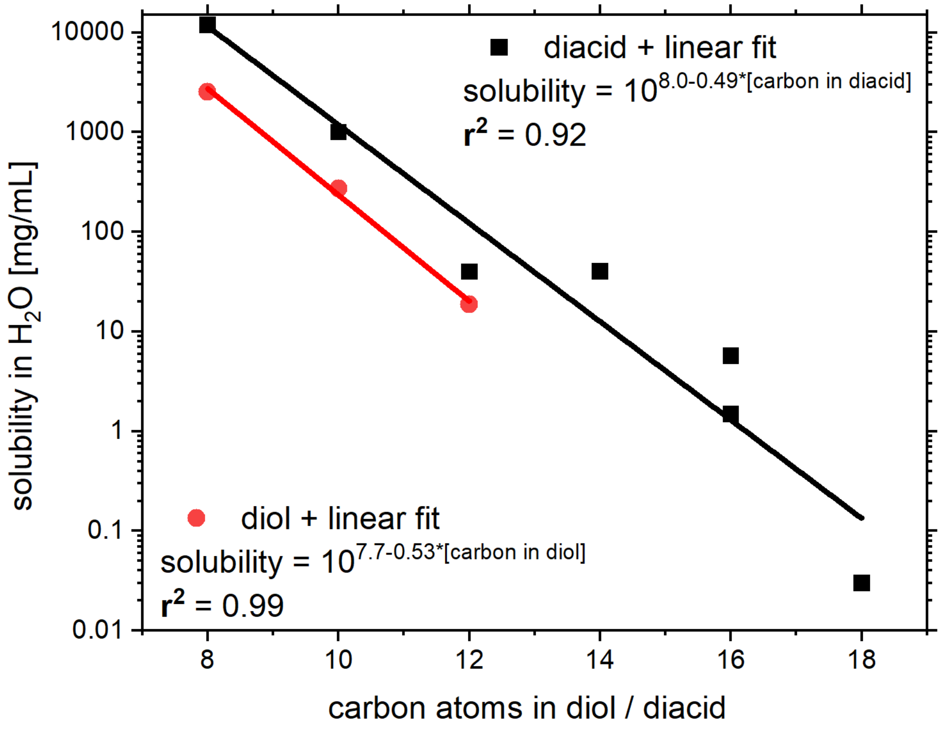

Figure S29: Linear fits of the solubility of aliphatic diacids and diols vs. the carbon number in their chains.

References

(1) Stempfle, F.; Quinzler, D.; Heckler, I.; Mecking, S. Long-Chain Linear C 19 and C 23 Monomers and Polycondensates from Unsaturated Fatty Acid Esters. *Macromolecules* **2011**, *44* (11), 4159–4166. https://doi.org/10.1021/ma200627e.

(2) Häußler, M.; Eck, M.; Rothauer, D.; Mecking, S. Closed-Loop Recycling of Polyethylene-like Materials. *Nature* **2021**, *590* (7846), 423–427. https://doi.org/10.1038/s41586-020-03149-9.

(3) Eck, M.; Schwab, S. T.; Nelson, T. F.; Wurst, K.; Iberl, S.; Schleheck, D.; Link, C.; Battagliarin, G.; Mecking, S. Biodegradable High-Density Polyethylene-like Material. *Angew. Chem., Int. Ed.* **2023**, *135* (6). https://doi.org/10.1002/ange.202213438.

(4) William Henry Bragg, William Lawrence Bragg. The Reflection of X-Rays by Crystals. *Proc. R. Soc. Lond. A* **1913**, *88* (605), 428–438. https://doi.org/10.1098/rspa.1913.0040.

(5) Holzwarth, U.; Gibson, N. The Scherrer Equation versus the "Debye-Scherrer Equation." *Nat. Nanotechnol.* **2011**, *6* (9), 534–534. https://doi.org/10.1038/nnano.2011.145.

(6) Fowkes, F. M. Attractive Forces at Interfaces. *Ind. Eng. Chem.* **1964**, *56* (12), 40–52. https://doi.org/10.1021/ie50660a008.

(7) Ribitsch, D.; Herrero Acero, E.; Greimel, K.; Dellacher, A.; Zitzenbacher, S.; Marold, A.; Rodriguez, R. D.; Steinkellner, G.; Gruber, K.; Schwab, H.; Guebitz, G. M. A New Esterase from Thermobifida Halotolerans Hydrolyses Polyethylene Terephthalate (PET) and Polylactic Acid (PLA). *Polymers* **2012**, *4* (1), 617–629. https://doi.org/10.3390/polym4010617.

(8) Chowhan, Z. T. pH-Solubility Profiles or Organic Carboxylic Acids and Their Salts. *J. Pharm. Sci.* **1978**, *67* (9), 1257–1260. https://doi.org/10.1002/jps.2600670918.

(9) PubChem. *Suberic acid*. https://pubchem.ncbi.nlm.nih.gov/compound/10457 (accessed 2024-03-05).

(10) *1,8-octane diol, 629-41-4*. http://www.thegoodscentscompany.com/data/rw1150351.html (accessed 2024-03-05).

(11). https://elevance.com/product/elevance-inherent-c18-diacid/?prod-id=426&cat-id=polymer_building_blocks-13 (Accessed 2023-08-21).

(12) *Registration Dossier - ECHA*. https://echa.europa.eu/registration-dossier/-/registered-dossier/19334/7/2/1 (accessed 2024-03-05).

(13) *Brief Profile - ECHA*. https://echa.europa.eu/brief-profile/-/briefprofile/100.024.667 (accessed 2024-03-05).

(14) *Brief Profile - ECHA C14*. https://echa.europa.eu/brief-profile/-/briefprofile/100.011.342 (accessed 2024-03-05).

(15) *Showing metabocard for Hexadecanedioic acid (HMDB0000672)*. https://hmdb.ca/metabolites/HMDB0000672 (accessed 2024-03-05).
